# Safe To Walk? Neighborhood Safety and Physical Activity Among Public Housing Residents

**DOI:** 10.1371/journal.pmed.0040306

**Published:** 2007-10-23

**Authors:** Gary G Bennett, Lorna H McNeill, Kathleen Y Wolin, Dustin T Duncan, Elaine Puleo, Karen M Emmons

**Affiliations:** 1 Department of Society, Human Development and Health, Harvard School of Public Health, Boston, Massachusetts, United States of America; 2 Center for Community-Based Research, Dana-Farber Cancer Institute, Boston, Massachusetts, United States of America; 3 Department of Health Disparities Research, University of Texas M. D. Anderson Cancer Center, Houston, Texas, United States of America; 4 Department of Surgery, Washington University School of Medicine, St Louis, Missouri, United States of America; 5 Department of Biostatistics and Epidemiology, University of Massachusetts, Amherst, Massachusetts, United States of America; Institut de Médecine Sociale et Preventive, Switzerland

## Abstract

**Background:**

Despite its health benefits, physical inactivity is pervasive, particularly among those living in lower-income urban communities. In such settings, neighborhood safety may impact willingness to be regularly physically active. We examined the association of perceived neighborhood safety with pedometer-determined physical activity and physical activity self-efficacy.

**Methods and Findings:**

Participants were 1,180 predominantly racial/ethnic minority adults recruited from 12 urban low-income housing complexes in metropolitan Boston. Participants completed a 5-d pedometer data-collection protocol and self-reported their perceptions of neighborhood safety and self-efficacy (i.e., confidence in the ability to be physically active). Gender-stratified bivariate and multivariable random effects models were estimated to account for within-site clustering. Most participants reported feeling safe during the day, while just over one-third (36%) felt safe at night. We found no association between daytime safety reports and physical activity among both men and women. There was also no association between night-time safety reports and physical activity among men (*p* = 0.23) but women who reported feeling unsafe (versus safe) at night showed significantly fewer steps per day (4,302 versus 5,178, *p* = 0.01). Perceiving one's neighborhood as unsafe during the day was associated with significantly lower odds of having high physical activity self-efficacy among both men (OR 0.40, *p* = 0.01) and women (OR 0.68, *p* = 0.02).

**Conclusions:**

Residing in a neighborhood that is perceived to be unsafe at night is a barrier to regular physical activity among individuals, especially women, living in urban low-income housing. Feeling unsafe may also diminish confidence in the ability to be more physically active. Both of these factors may limit the effectiveness of physical activity promotion strategies delivered in similar settings.

## Abstract 2

Garry Bennett and colleagues measured exercise levels and obtained opinions on neighborhood safety. They concluded that residing in a neighborhood perceived to be unsafe at night is a barrier to regular physical activity.

## Editors' Summary

**Background.:**

Nowadays we are all encouraged to do more physical activity, as it has been shown that inactivity increases the risk of many medical conditions, including obesity, heart attacks, and strokes. Inactive people die younger. Previous research has shown that people on a low income and those from ethnic or racial minorities have the lowest activity levels. There are, however, many barriers to being active. It has been claimed that people who live in neighborhoods that are unsafe face particular difficulties. They might want to walk, cycle, or take other forms of outdoor exercise near their home, but they fear they would be injured as a result of a violent attack. It is usually the poorest members of society who live in unsafe areas. It is also known that those poor people who belong to minority racial or ethnic groups are particularly likely to feel unsafe.

**Why Was This Study Done?:**

The researchers who did this study wanted to find out whether people in a low-income urban area in the US considered themselves to be unsafe in their neighborhood, and how much physical activity they took part in. Their aim was to establish whether there was an association between the perceived safety level and the amount of activity taken, or if the two were unrelated. Other researchers have tried to look for such an association before, but they have usually relied on how much activity people say they remember taking, not on the actual measured amount. The results from such research have been very varied and inconclusive.

**What Did the Researchers Do and Find?:**

Working in one low-income district of one US city (Boston), they found over 1,000 people to participate in their study, most of whom were from ethnic minorities. They asked them the question: “How safe do you feel walking alone in your neighborhood?” Response options included “safe,” “a little unsafe,” and “unsafe.” The same question was asked about walking alone in the daylight and walking alone after dark. The people in the study also agreed to wear a pedometer for five days. This instrument measures the number of steps that the wearer takes. It is thus a much more accurate way of finding out about activity levels than asking people how much activity they think they have engaged in.

Four out of five people said they did feel safe during the day, but there was no association between daytime safety and physical activity. This was the case for both men and women. Two-thirds of the people in the study felt unsafe in the night-time. There was no association between perceived night-time safety and physical activity among men, but women who reported feeling unsafe at night took around 1,000 fewer steps per day than other women. That amounts to around 20% less physical activity.

**What Do These Findings Mean?:**

Even the women who felt safe at night were only taking around 5,000 steps per day, around half of what the US Surgeon General recommends for good health. So all the women in the study would benefit from more physical activity. However, the much lower amount of activity of the women who felt unsafe does suggest that a perceived lack of safety is an important factor, which could increase the risks to their health. It is interesting that the association between perceived safety and activity was not found in men and only applied to night-time safety. However, the authors argue that their findings provide preliminary evidence that perceived low neighborhood safety may serve as a barrier to physical activity in low-income areas. They discuss in the article the need for further research.

**Additional Information.:**

Please access these Web sites via the online version of this summary at http://dx.doi.org/10.1371/journal.pmed.0040306.

• The UK National Health Service publishes the online NHS Direct Health Encyclopedia, which describes the benefits of exercise and gives recommendations

• Information on exercise may also be found on MedlinePlus, a service of the US National Library of Medicine and the National Institutes of Health

## Introduction

The benefits of regular physical activity have been frequently documented [1–3]. Physical activity is associated with reduced risk of numerous chronic conditions and premature mortality [4]. Indeed, up to 20% of chronic disease mortality may be attributed to physical inactivity [1]. Although some data suggest that the prevalence of physical inactivity is declining [5–7], sedentary behavior remains pervasive. The highest levels of physical inactivity are found among racial/ethnic minorities and those of lower socioeconomic position (SEP) [5,7–15], despite widespread recognition of the health benefits of regular physical activity in these groups [16].

Neighborhood factors such as perceived safety have received increasing attention as barriers to physical activity. Perceptions of neighborhood safety may be particularly salient among those residents in lower-income urban settings who are from racial or ethnic minority groups. Indeed, racial/ethnic minorities and those of lower SEP are the most likely to rate their neighborhoods as unsafe [17–19]. While many have hypothesized an inverse association between the perception of unsafe surroundings and physical activity, empirical support for the association has been inconsistent [20]. A number of studies, for example, have found support for an inverse relation between perceived neighborhood safety and physical activity in adults [21–36] and children [37–42]; however, numerous studies have shown no relation [9,19,33,43–57].

A major limitation of previous studies investigating the association between neighborhood safety and physical activity has been, with few exceptions [50], the use of self-reported physical activity measures. The validity of these measures may be compromised [58], because few self-report measures are designed to assess physical activity accumulated through routine, nonleisure activities (e.g., domestic, occupational, and transportation), which may account for a greater proportion of total physical activity among those of lower SEP [12,59].

Individuals in urban settings may have a greater likelihood of engaging in certain physical activities (particularly walking) for transportation purposes (e.g., walking to work, school, or a bus stop). While walking is the most frequently adopted type of regular physical activity [59,60], particularly among some racial/ethnic minority groups [59], it is also among the least reliably recalled activity types [59,61]. Pedometers yield objective physical activity measures that may minimize some of the challenges associated with self-report [62]. Recent studies [62–64] have demonstrated the utility of pedometers in studies of physical activity among racial/ethnic minority and low-income populations.

Nearly 1.5 million US households, with a disproportionate number of racial/ethnic minorities, currently reside in public housing (i.e., affordable housing for people of low income, subsidized by the federal government). Little research has examined the potential salience of perceived neighborhood safety for physical activity behaviors in the low-income housing setting. As such, the primary goal of the present investigation was to evaluate the association between perceived neighborhood safety and pedometer-determined physical activity among a sample of predominantly racial/ethnic minority adults residing in low-income, public housing. We were particularly interested in examining whether the studied associations varied by gender, an important physical activity correlate. In addition, given the available data detailing the importance of self-efficacy in predicting physical activity intervention uptake, a secondary aim of the study was to examine the relation between perceived neighborhood safety and physical activity self-efficacy.

## Methods

This study used a randomized cluster design with 12 urban public housing communities in metropolitan Boston as the primary sampling units. The housing sites involved range in size and layout from high-rise apartment buildings to more dispersed townhouse-style complexes. Secondary sampling units were individuals within the sites. Unequal probability sampling across housing sites was employed due to the varying sizes of the sites. In half of the sites (with populations less than 300 persons), the full population was sampled, and in the remaining sites (with populations greater than 300 persons), sampling was conducted to obtain an approximate 35% sample with a minimum of 250 individuals per site.

Participant recruitment began with housing site representatives sending letters announcing the study to their eligible residents. Eligibility criteria for the study survey included: (1) residence in the housing community, (2) age at least 18 y, and (3) fluency in English or Spanish. Residents were provided the ability to opt-out of the study by contacting either a housing site representative or member of the research staff. An initial sample size of 3,368 individuals was drawn. Of them, 747 (20%) were deemed ineligible, leaving an eligible sample population of 2,941 individuals. Of these, 828 (28%) refused participation and 559 (19%) were never reached. Enrollment and baseline surveys were obtained on 1,554 participants. This yielded an overall 53% response rate, which ranged from a low of 34% to a high of 92% across the housing sites. As explained below, a further reduction related to compliance with the pedometer sampling protocol reduced the response rate to 40% overall for this study. Participants provided informed consent and completed the interviewer-administered survey in either English or Spanish. The Human Subjects Committee at the Harvard School of Public Health approved the study protocol.

### Pedometer Sampling Protocol

The pedometer protocol has been described in greater detail elsewhere [62]. Briefly, following completion of the baseline survey, a member of the study staff oriented participants to the sampling protocol and provided each person with a kit, containing the pedometer (with lanyard to secure the pedometer), sampling log, instructions (with photos), and a storage container. Research staff explained the functions of the pedometer, reset it, and taped the pedometer shut, blinding participants to the step count. Staff demonstrated proper pedometer placement and use of the lanyard, and reviewed instructions for completion of the sampling log.

Participants were instructed to wear a pedometer for five days (beginning with the day of survey administration) at all times except while bathing, showering, swimming, or sleeping. Pedometer sampling began on all seven days of the week, and with the exception of those starting on Mondays, included at least one weekend day.

Participants wore the pedometer from the time they awakened until going to bed. After the fifth day, participants were asked to remove the pedometer and place it in the provided storage container before going to bed; the pedometer was not to be removed from the container until it was returned to study staff (which was typically on the same or next day). The pedometers were taped shut so that participants could not see the step count, and they were asked not to open them. Upon receipt of the pedometer, staff checked for signs of tampering, and immediately recorded the accumulated steps.

The study pedometers (Yamax SW200) demonstrate high concordance with accelerometers under both laboratory conditions and in field settings [65,66]. Before being provided to participants, all pedometers were fully tested, using Tudor-Locke's method, to ensure that they were fully operational [60]. Participants were provided a $25 grocery store card incentive upon completion of the data collection protocol.

### Perceived Neighborhood Safety

Two items, based on previously tested questions (with slight modifications to accommodate literacy concerns) were utilized to measure perceived neighborhood safety [67,68]. For both “daytime” and “night-time,” participants were asked: “… how safe do you feel walking alone in your neighborhood?” Response options included, “safe,” “a little unsafe,” and “unsafe.” For analysis purposes, we combined the response categories of “a little unsafe” and “unsafe” for daytime safety due to the small number of responses in the latter category. These questions are designed to capture global perceptions of neighborhood safety and as such, may reflect views on a variety of factors (e.g. crime, traffic, green space, etc.).

### Physical Activity Self-Efficacy

Physical activity self-efficacy was assessed using a modification of the Self-Efficacy and Exercise Habits Survey [69]. Four items were selected, representing each of the two domains (“sticking to it” and “making time for exercise”) from the original scale; a four-point Likert response scale, ranging from “very sure” to “very unsure” was used. Cronbach's alpha for the resulting measure was 0.80.

### Sociodemographic Characteristics

Participants self-reported their age in years; we subsequently coded age into 10-y intervals. Participants were asked to report their race/ethnicity as: black, white, Hispanic, Asian, American Indian, or other. Participants were permitted to select more than one option; those who selected Hispanic were coded as such, regardless of other options selected. Participants choosing more than one of the other five race/ethnicity options were assigned to a “mixed race/ethnicity” category. Participants reported their highest level of educational attainment, which was collapsed into three levels due to small numbers (less than high school, high school or vocational school, any post-high school education). Participants' current employment status was grouped into four levels: working full-time, working part-time, disabled from working, not working—including retired and homemaker. Body mass index (BMI; kg/m^2^) was calculated from either self-reported or measured height/weight. All analyses used BMI as a continuous variable.

### Statistical Analysis

All study participants were enrolled in the physical activity protocol unless deemed ineligible because they were either not ambulatory or their literacy levels were too low to complete the sampling log (*n* = 59, 4%). We excluded from our analyses participants who did not wear the pedometer for at least 3 d (*n* = 256, 16%), those who returned broken pedometers or had violated the study protocol (*n* = 22, 1%), those who became incapacitated during the 5-d study period or were further deemed not ambulatory or of low literacy (*n* = 9, <1%), and those whose log data were incomplete (*n* = 23, 1%). An additional five (< 1%) were deleted from the analysis dataset because their pedometer readings averaged less than 1 step per day. These reductions left 1,180 (76%) participants. We used gender-stratified random effects models and controlled for clustering of participants within housing sites. In analyses predicting the physical activity self-efficacy outcome, odds ratios (ORs) represent the odds of one having high physical activity self-efficacy. Gender-stratified age-adjusted bivariate models and multivariable models adjusting for age, BMI, race/ethnicity, and employment status are presented. For all analyses, based on the cluster design, data are weighted to the population size within each housing site (with a total weighted size of 1,735). Analyses were conducted using SUDAAN version 9.01 and SAS version 9.1 statistical software for clustered data.

## Results

As shown in Table 1, the study sample was predominately female (73.2% weighted) and was largely composed of racial/ethnic minorities; most participants identified themselves as Hispanic (42.1% weighted) or black (43.6% weighted). Most of the residents were either not currently working or were disabled (59.6% weighted). Average age was approximately 49 y and mean BMI was 30.0 kg/m^2^. Participants accumulated an average of 5,649 steps per day (steps/d) (range 500-2000). There was a striking difference between perceptions of neighborhood safety by time of day. More than 80% (weighted) of the respondents reported feeling safe during the daytime, whereas only 37% (weighted) reported feeling safe at night-time. As expected, males tended to report feeling safer than females at both times of day.

**Table 1:**
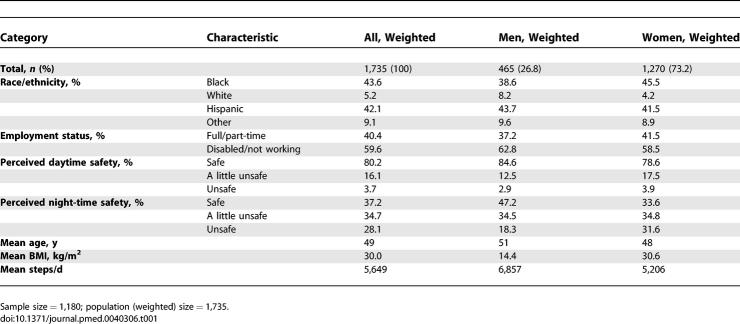
Demographic Characteristics of Study Sample

Tables 2 and 3 display the mean steps/d in regression equations predicting steps/d where the independent variable of interest is perceived neighborhood safety. For night-time reports (Table 2) of perceived neighborhood safety, we saw no association with steps/d among men. For women, however, feeling unsafe was significantly associated with steps/d in both age-adjusted bivariate and multivariable-adjusted models; women feeling unsafe at night had 1,107 fewer steps/d than those who identified their neighborhoods as safe in the multivariable model. During the daytime (Table 3), although reporting one's neighborhood as unsafe was associated with significantly fewer steps/d for both men and women in age-adjusted bivariate and multivariable-adjusted models (unpublished data), this association did not remain statistically significant when response categories were collapsed (i.e., “a little unsafe” combined with “unsafe”).

**Table 2:**
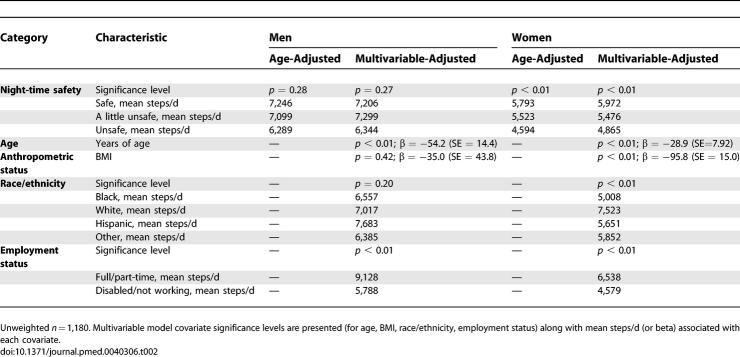
Perceived Night-time Neighborhood Safety and Steps Per Day

**Table 3:**
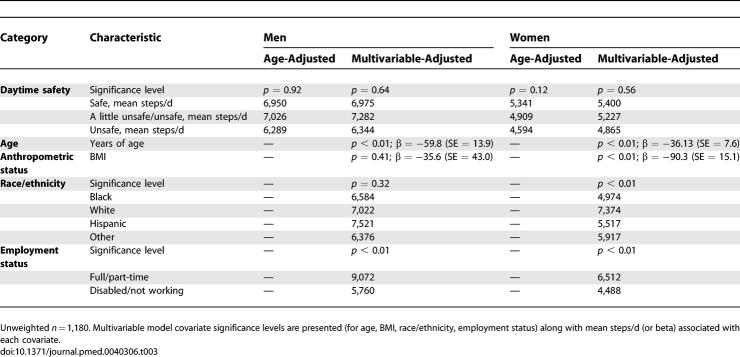
Perceived Daytime Neighborhood Safety and Steps Per Day

Tables 4 and 5 display ORs from logistic regression models predicting high physical activity self-efficacy (restricted to the 1,180 participants who had physical activity data). Compared to those reporting safe neighborhood surroundings at night (Table 4), men who reported feeling a little unsafe or unsafe were significantly less likely to have high physical activity self-efficacy. This relation did not hold true for women. In multivariable-adjusted models, men reporting feeling a little unsafe at night were 51% less likely to have high physical activity self-efficacy than those who felt safe. Compared to those reporting safe neighborhood surroundings in the daytime (Table 5), in multivariable models, there was significant variation in physical activity self-efficacy for both men and women. Men who felt a little unsafe or not at all safe were 51% less likely to have high physical activity self-efficacy, whereas women who felt similarly were 32% less likely.

**Table 4:**
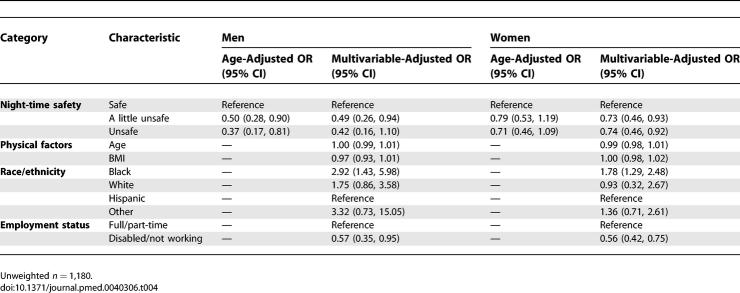
Perceived Night-Time Neighborhood Safety and Physical Activity Self-Efficacy

**Table 5:**
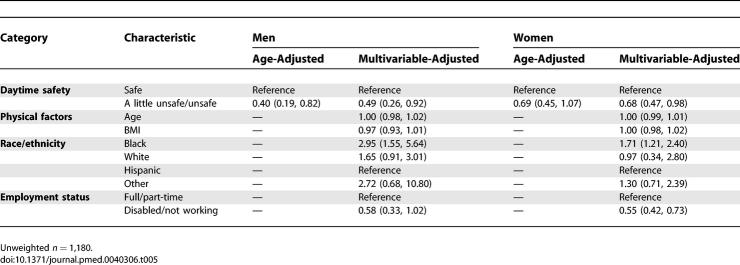
Perceived Daytime Neighborhood Safety and Physical Activity Self-Efficacy, OR (95% CI)

## Discussion

Boston, like other major cities in the United States and European Union (an intergovernmental union of 27 European nations) has experienced an increase in violent crime after historic lows since the mid-1990s; however, few studies have explored how perceptions of perceived safety might impact physical activity practices in the communities most affected by this pressing social exposure. In contrast to previous studies, our work highlights the importance of considering neighborhood safety at night-time as a possible barrier to physical activity among women. We found that women reporting their neighborhoods as unsafe during night-time hours had nearly 1,100 fewer steps/d than those who viewed their surroundings as safe. We consider this effect sizeable relative to the studied demographic predictors and considering the average steps/d (5,206) among female participants. This average itself is clearly short of the 10,000 steps/d threshold that is roughly comparable to the number of steps/d necessary to meet the consensus US Centers for Disease Control/American College of Sports Medicine national physical activity guidelines [70–72]. Thus the effect on steps/d of rating one's neighborhood as unsafe accounts for approximately one-fifth of steps/d in our study and one-tenth of the nationally recommended steps/d for women in the sample.

There was however, no consistent dose–response relation of safety with physical activity. We also found no association of daytime safety ratings with steps/d for either men or women. This was somewhat surprising because we suspected that daytime safety reports may be more reflective of the overall dangerousness of the neighborhood. It is also possible that reports of unsafe daytime surroundings may differentially inhibit physical activity by gender; for women, transportation, occupational, and domestic activities may require daytime physical activity, independent of safety concerns [73]. It is also possible that we may have seen no association among those who work in a different neighborhood from the one of their residence; for such individuals, neighborhood ratings of safety may not impact total physical activity levels.

We also found that feeling unsafe in one's neighborhood was associated with decreased confidence in the ability to be physically active. Indeed, our findings showed a lower likelihood of high self-efficacy among those perceiving their neighborhood as either unsafe or a little unsafe during the day, and among women rating their neighborhoods as unsafe at night. Self-efficacy has long been identified as an important mediator of health behavior change, and is a consistently strong predictor of physical activity intervention uptake [74]. To our knowledge, no studies have examined the influence of perceived neighborhood safety on physical activity self-efficacy (though some work has examined other aspects of self-efficacy [44,48]).

That neighborhood safety impacts not only physical activity, but also self-efficacy suggests that individually oriented physical activity promotion strategies directed to similar populations may be ineffective without considering strategies to assist individuals to identify safe, convenient, and comfortable contexts in which to be physically active. Of course, these findings also highlight the importance of considering policy-level strategies, such as local police involvement and community efforts to reduce crime. While we advocate for the use of green space, parks, and recreational facilities for physical activity (which are actually quite prevalent in many of the target communities), these are also locations where (in Boston and many other urban areas), violent crime frequently occurs.

### Study Strengths and Limitations

Several considerations may limit interpretations drawn from these data. First, these data were cross-sectional and as such, provide no direct evidence of a physical activity benefit associated with improving perceptions of neighborhood safety. As mentioned, we did not assess specific domains of neighborhood safety, nor did we use objective measures of neighborhood safety (e.g., crime statistics), because we were specifically interested in examining individual perceptions of safety; nevertheless, each are likely to be differentially associated with physical activity outcomes. We have focused our discussion primarily on crime for several reasons; our formative research in the housing setting strongly suggests that neighborhood violence is the primary determinant of concerns about neighborhood safety. Importantly, this study was conducted at a time when neighborhood violence in the target communities was in the midst of a dramatic increase relative to previous years.

Our sampling strategy is supported by the results of a recent validation study [75] that found that any three days (weekday or weekend) are sufficient to reliably estimate physical activity performed in a free-living week. A higher response rate would have been desirable; challenges experienced in the initial two housing sites (during the study's startup phase) most negatively influenced the estimate. However, the overall response rate (53%) should be considered in the context of the many challenges inherent in conducting research in this setting and the complexity of the physical activity assessment protocol. Nevertheless, generalizability of the findings is constrained to those individuals residing in comparable communities and should be considered in light of our study response rates.

Ideally, we would have measured height and weight among all participants, but this was not feasible in some housing sites (and some participants refused), so self-reported height and weight was used. We found that adjusting for BMI measurement did not influence our results. Like many similar studies, we lacked the ability to fully adjust for the full range of possible factors that might be potentially associated with physical activity in the low income housing context.

Finally, a key strength is that, to our knowledge this is the largest study examining perceived neighborhood safety to include pedometer-determined physical activity. Use of an objective measure of total physical activity is an important advance of the present study. Others and we have previously shown that those in low SEP may accumulate a greater proportion of their daily physical activity through nonleisure domains [62,76]. As such, use of pedometers may provide a sensitive measure of total physical activity among lower income populations.

### Conclusions

Urban areas are highly walkable environments with sidewalks and a variety of land-mix uses. Neighborhood streets are the most common venue for walking [77] and walking as a behavior is increasing among racial/ethnic minority groups [78]; walking for exercise may be particularly important in low-income neighborhoods because it requires few financial resources. Our data provide preliminary, albeit cross-sectional, evidence that perceived neighborhood safety may serve as a barrier to physical activity in low-income settings. Prospective studies examining these associations are sorely needed. Such studies would serve well to examine both objective safety indicators and individuals' perceptions. Whether individual perceptions modify the influence of objective indicators on physical activity is an important area of study. Evidence documenting a causal relation between perceived safety and physical activity would lend additional support to policy efforts (e.g., enhancing the attractiveness of urban, lower income neighborhoods, revitalizing neighborhood watch programs to monitor criminal activity, working with local governments to install traffic-calming devices, and liaising with police to enhance the protection of parks and recreation facilities) designed to create environments that are suitable for physical activity.

## Abbreviations:

BMI: body mass index
SEP: socioeconomic position
